# [Corrigendum] Eupafolin inhibits breast cancer cell proliferation and induces apoptosis by inhibiting the PI3K/Akt/mTOR pathway

**DOI:** 10.3892/ol.2026.15635

**Published:** 2026-05-05

**Authors:** Jiahui Wei, Xuefeng Zhang, Huihao Pan, Song He, Bao Yuan, Qing Liu, Jiabao Zhang, Yu Ding

Oncol Lett 21: 332, 2021; DOI: 10.3892/ol.2021.12593

Subsequently to the publication of the above paper, an interested reader drew to the authors’ attention that the data panels showing the migration and invasion assay experiments for treatment with 25 μM Eupafolin in Fig. 2C on p. 4 contained a small overlapping section, such that data which were intended to show the results from differently performed experiments had apparently been derived from the same original source.

The authors explained that, owing to the time that has elapsed since this paper was published, they were unable to locate the raw data underlying the images shown in Fig. 2C, although they were confident that this figure had been inadvertently assembled incorrectly. The Editor of *Oncology Letters* requested that the authors repeat the affected experiments, and they confirmed that they were willing and able to undertake this extra work. The revised version of Fig. 2, now showing new data from one of the repeated experiments in Fig. 2C, is shown on the next page. Note that the revisions made to this figure do not affect the overall conclusions reported in the paper. The authors are grateful to the Editor for allowing them the opportunity to publish this Corrigendum, and apologize to the readership for any inconvenience caused.

## Figures and Tables

**Figure 2.**
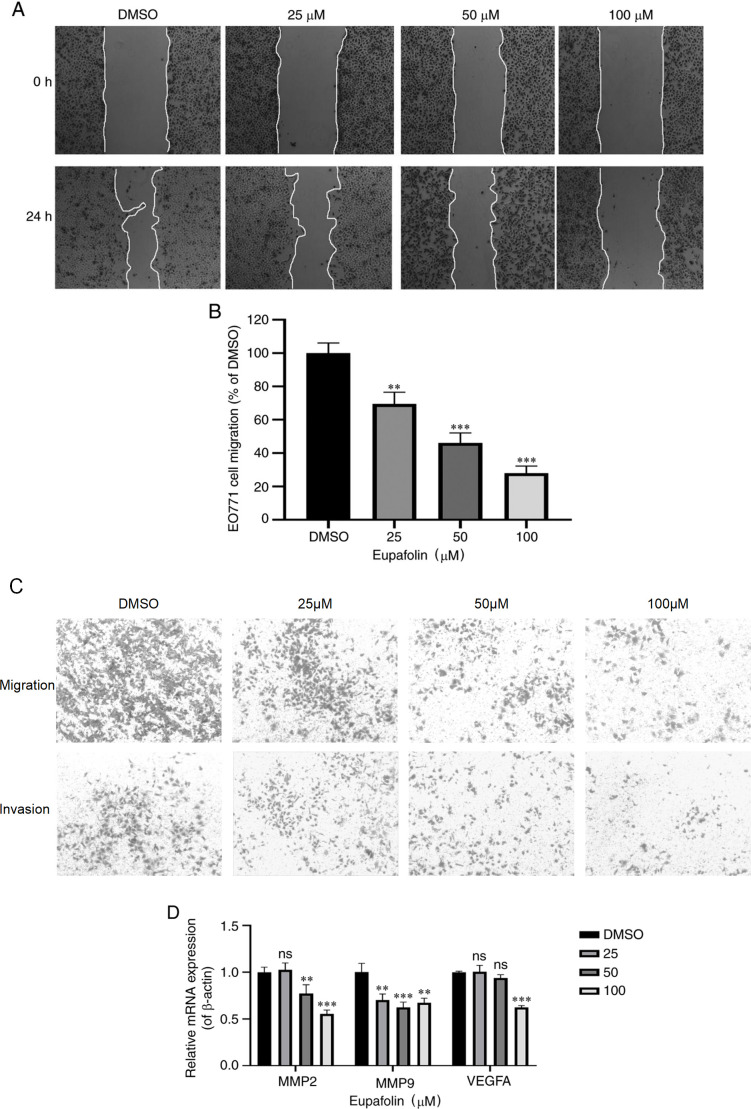
Eupafolin attenuates the invasion and migration of EO771 cells. After seeding EO771 cells in a 6-well plate and treating them with different concentrations of Eupafolin for 24 h, the migration distance of the cells was measured, the proportion of invaded cells was calculated, and RNA was extracted from cells to determine the expression of associated genes using RT-qPCR. (A and B) 24 h after Eupafolin treatment, the healing effect of EO771 cells was determined using the scratch test. (C) The ratio of invasion and migration of EO771 cells was further determined using a transwell assay. (D) EO771 cells were harvested at 24 h post treatment for RT-qPCR to determine the mRNA expression of MMP2, MMP9 and VEGF-A. Data are expressed as the mean ± SD. ns, P>0.05; **P<0.01; ***P<0.001. MMP, matrix metallopeptidase; RT-qPCR, reverse transcription-quantitative PCR; VEGF, vascular endothelial growth factor.

